# Improvement of Rheological, Textural and Sensorial Properties of Dough and Enriched Cakes by Addition of *Cucumis melo* L. Peels Powder

**DOI:** 10.1002/fsn3.71313

**Published:** 2026-06-02

**Authors:** Sana Mallek‐Ayadi, Neila Bahloul

**Affiliations:** ^1^ Laboratory of Applied Fluids Mechanics, Process Engineering and Environment, Research Group of Agri‐Food Processing Engineering, National School of Engineers of Sfax University of Sfax Sfax Tunisia

**Keywords:** cake formulation, dough rheology, melon peel flour, sensory attributes, textural properties

## Abstract

Melon peel is a by‐product of the food processing industry. It contains fibers, polyphenols and proteins which can enhance human health. The present research aims to evaluate the effect of melon peel powder (MPP) as a partial substitute for wheat powder, on dough characteristics and cake quality. Results demonstrated that melon peel powder exhibited interesting functional properties such as water holding and oil binding capacities. Moreover, the integration of MPP at diverse levels (10%, 20%, and 30%) was found to affect the rheological and textural features of the doughs. Indeed, it allowed for an increase in dough tenacity (*P*) and deformation energy (*W*) and a decrease in dough extensibility (*L*). Textural properties revealed that the incorporation of MPP led to an increase in cohesion, adhesion and stickiness of the dough and a clear decrease in dough hardness. The partial replacement of wheat flour with MPP substantially enhanced the texture profile of MPP‐enriched cakes, as evidenced by the reduction in cake hardness and chewiness. The MPP‐enriched cakes up to 10% maintained the sensory attributes in comparison to the control cake. These findings proved the potential of using MPP to produce fiber‐rich cakes with enhanced textural and sensorial properties.

## Graphical abstract

Melon peel powder (MPP) exhibited interesting functional properties as water holding and oil binding capacities. The integration of MPP at diverse levels (10%, 20%, and 30%) was found to affect the rheological and textural features of the doughs. The partial replacement of wheat flour with MPP enhanced substantially the texture profile of MPP‐enriched cakes, as evidenced by the reduction of cake hardness and chewiness.

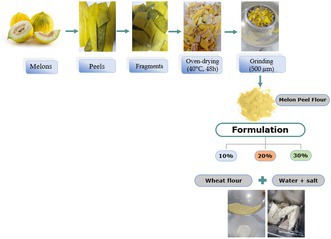

## 1 Introduction

In recent years, food industries have started to use flours with nutritional value in the formulation of baked foods. The leftovers from fruit and vegetable processing are regarded as low‐cost sources of beneficial and nourishing ingredients (Gômez and Martinez 2018; Bravo‐Nuňez and Gômez 2021). Significant amounts of fruit peel waste (often 49% of the entire fruit weight) are generated by the fruit processing industry (Parafati et al. 2020), however, this considerable quantity of by‐products is often rejected. As a result, studies on the use of fruit powders in baking products are notably widespread nowadays. For instance, biscuits and cakes are among the most consumed foodstuffs that are daily consumed due to their pleasant taste and affordable cost (Nyam et al. 2013).

Melon ( *Cucumis melo* L.) is cultivated in several countries with temperate climates across Asia Africa, Europe and America (Vella et al. 2023). Melon is one of the most commonly expanded fruits throughout the world and their peels are among industrial processing by‐products. Melon peels are characterized by their high contents of fibers, minerals, amino acids, and proteins, as well as antioxidants and other bioactive molecules with healthy effects on disease prevention (Mallek‐Ayadi et al. 2017; Hussain et al. 2024). The fruit processing industry values the comestible part of the melon fruit, but seeds and peels are often discarded and not valorized, while they can be used as functional ingredients after conversion into flour. Regrettably, data regarding the incorporation of melon peel flour in cake formulation are scarce in the literature. Therefore, efforts have been made to make better use of these by‐products as functional ingredients and potential new supplies of bioactive components (Parafati et al. 2020). Thus, the current study was undertaken: (i) to assess the chemical attributes and functional properties of melon peel powder (MPP), (ii) to evaluate the influence of MPP addition on the alveographic characteristics of enriched doughs, and (iii) to assess the effects of melon peel powder on the textural and sensorial attributes of enriched cakes.

## 2 Materials and Methods

### 2.1 Vegetable Raw Material

Melon fruits derived from the Maazoun variety were acquired from Sfax governate (Tunisia) in June 2024. Fruit ripeness was assessed based on the light‐green color of the melon peel. At once, matured melon fruits were cleaned of any adhering residue and stored in the laboratory until use.

### 2.2 Melon Peel Powder Preparation

Melon peels were removed from the fruits, washed and chopped into tiny fragments. They were dehydrated at 40°C in an oven for 48 h; then, they were ground using an electric mill. The regular particle size of peel powder was 500 μm. The obtained melon peel powder (MPP) was stored for further analyses.

### 2.3 Chemical Composition of Melon Peel Powder

Fat, protein, moisture and ash of melon peel powder were carried out in accordance with the Association of Official Agricultural Chemists method (AOAC 2002). Moisture was determined by drying samples in a laboratory oven regulated at 105°C up to constant weight. Protein content of melon peel flour was determined by the Kjeldahl method. The protein amount was calculated using a nitrogen conversion factor of 6.25 (Ortiz et al. 2006). The fat amount was determined by following the method of Soxhlet extraction using hexane as a solvent. Values were expressed as the percentage of dry matter. Furthermore, ash analysis was achieved by incineration of samples in a muffle furnace at 550°C for 4 h. The carbohydrate amount was assessed by calculating the difference of mean values: 100 − (sum of proportions of ash, protein, and fat) (Lima et al. 2014). Total dietary fiber was resolved using the AOAC enzymatic gravimetric method (Prosky et al. 1988).

### 2.4 Functional Properties

#### 2.4.1 Bulk Density

Bulk density was achieved as reported by Kaur and Singh (2005) and expressed as grams per milliliter. Melon peel flour was filled into a measuring cylinder (10 mL). The cylinder bottom was tapped on a laboratory bench several times after filling to the 10 mL mark.

#### 2.4.2 Swelling Water Capacity

The bed volume technique was adopted to calculate swelling capacity (Robertson et al. 2000). First, the samples were precisely weighed and then they were transferred into a standardized cylinder. After that, 10 mL of distilled water containing 0.02% sodium azide was added. The measurement cylinders were kept to stand for 18 h at room temperature. The bed volume was then defined as volume per gram sample.

#### 2.4.3 Water Solubility Index

The method of Anderson et al. (1969) was followed to calculate the water solubility index (WSI) of peel powder. The sample (2.50 g) was distributed in 30 mL of distilled water and cooked for 15 min in a water bath at 90°C. Next, the heated peel was centrifuged for 10 min at 3000 *g*. The supernatant was decanted to measure its solid content into a tarred evaporating dish. The dried solids' weight increased when the supernatant was evaporated at 110°C overnight. The following formula was applied to calculate the WSI:

$$\mathrm{WSI}\;\left(\%\right)=\left({\text{Weight}}_{\text{dissolved solids}}/{\text{Weight}}_{\mathrm{dry}\;\text{solids}}\right)\times 100$$

#### 2.4.4 Color Measurement

A calibrated colorimeter (CR‐410; Konica Minolta Inc.), was used to measure the CieLab coordinates (L*, a*, and b*) of melon peel powder, dough, and cakes. L* indicates lightness of the sample and ranges from black (0) to white (100). The a* value indicates green (negative value) or red–purple color (positive value). The b* value designates yellow (positive value) or blue color (negative value).

### 2.5 Oil Binding Capacity

The oil binding capacity was conducted according to the method described by Garau et al. (2007). Melon peel powder (±0.50 g) was mixed with sunflower oil (10 mL), centrifuged at 2000 *g* for 20 min and the excess supernatant was decanted. Oil binding capacity was expressed as g oil/g dry sample.

### 2.6 Dough Preparation

Four types of dough were produced: a control dough made of 100% wheat flour (400 g) (supplied by a Tunisian flour mill), and three doughs made by partial replacement of wheat flour with distinct melon peel powder proportions (10%, 20%, and 30%). All doughs were manufactured by mixing each flour with 240 mL distilled water and 1 g of salt (NaCl). The dough was prepared by mixing the flour and the water for approximately 10 min until a homogeneous dough was achieved. Each formulation was performed in triplicate.

#### 2.6.1 Alveograph Testing

Alveographic analyses of the dough were accomplished using an Alveograph Chopin (20 av. Marcellin Berthelo) and each dough sample (250 g) was obtained by mixing wheat and melon peel powder related to the adequate supplementation (10%, 20%, or 30%). The alveogram analysis allows measuring the tenacity (*P*), dough extensibility (*L*), deformation energy known as an index of baking strength (*W*) and the *P*/*L* ratio. These alveographic parameters are used to evaluate the dough quality.

#### 2.6.2 Textural Analysis

The dough samples were studied according to the texture profile analysis (TPA) technique by the use of a Texture Analyzer (LLOYD instruments, England) with a 1000 (N) load cell, and a 0.05 (N) detection range. A cylindrical probe (acrylic) was adopted to compress 50% of the sample of its original height (20 mm thickness) at a speed of 10 mm/s. The computer software furnished by Texture Technologies Corp. (Scarsdale, NY) and interfaced with the Texture Analyzer was used to analyze the data and control the instruments. Textural values of cohesion, adhesion, stickiness, and hardness were provided from the TPA graphic.

### 2.7 Baking Test

#### 2.7.1 Cake Preparation

Cake samples were manufactured from the following formula: 100 g sugar, 100 g flour blend, 25 g shortening, 120 g egg, 0.50 g baking powder, 1.50 g salt and 40 g sunflower oil. Cake batter was placed in a mixer wherein, the shortening, flour, baking powder, and salt were creamed together to get a tender cream. Additionally, sugar and eggs were well mixed until a semi‐firm foam appeared. The sponge cake ingredients were mixed for 3 min. After thoroughly whipping, the blend of egg‐sugar foam was subjected to blend with the creamed shortening and flour. Subsequently, the vegetable oil was supplemented in little proportions. After mixing, batters (200 g each) were placed in aluminum pans (internal dimensions: length 150 mm, width 50 mm, height 60 mm) and were baked in an electric oven at 180°C for 30 min. After baking, cakes were removed from the pan, left to cool for 60 min at room temperature, and packed into hermetically sealed bags to prevent drying, until evaluation. Baking tests were performed in triplicate for each variation.

#### 2.7.2 Physical Characteristics of Cakes

The cake density (*W*/*V*, g/cc) was calculated by the ratio between the weight of the cake and its volume. The volume (*V*, cc) of cake was measured using the rapeseed displacement method according to Acun et al. (2024). The weight of the cake was measured (*W*, g) by an analytical balance (Metteler AT 400) having a precision of 0.0001 g.

The textural profile of enriched cakes was determined by the use of a Texture Analyzer. The cake slices (2.5 cm thickness) were sited on the platform. A cylindrical probe (acrylic) was utilized, at a speed of 10 mm/s, to compress the samples 50% of their original height. Textural parameters (hardness, cohesion, adhesion, chewiness and breaking force) were determined. Color was measured using a Minolta CR‐410 Colorimeter (Konica Minolta Sensing Inc., Japan) with the D65 standard illuminant; results were expressed in the CIE L*a*b* color space. The color attributes of crumb and crust were checked for control cakes and cakes fortified with melon peel flour.

#### 2.7.3 Sensory Analysis

The sensorial features of cakes were achieved using 60 panelists. The participants were requested to assess the cakes for crumb color, crust color, odor, flavor, taste, tenderness, and overall quality (Sudha et al. 2007). The panelists used a 5‐point hedonic scale for each sensorial attribute.

### 2.8 Statistical Analysis

One‐way analysis of variance (ANOVA) was applied to assess significant differences (Tukey's test) using SPSS software (version 20.0; SPSS Inc., USA). The significance amount was defined as *p* < 0.05.

## 3 Results and Discussion

### 3.1 Chemical Composition and Functional Properties

Chemical analysis of melon peel powder (MPP) is summarized in Table 1A and demonstrates considerable amounts of protein (7.63%) and fiber (40.84%) which could enhance the nutritional value and technological potential of foodstuffs. The functional properties play a crucial role in the manufacturing of food products and determine whether the blends would be useful in bakery products. In this research, the functional properties of melon peels as bulk density, powder swelling, water solubility index, water holding capacity and oil binding capacity are determined (Table 1A). Bulk density is a measure of the heaviness of a flour sample (Oladele and Aina 2007). Melon peel flour is denser (0.51 g/mL) than watermelon peel powder accounting for 0.44 g/mL (Acun et al. 2025). The swelling power denotes the degree of water absorption of the starch granules in the flour (Julianti et al. 2017). The powder swelling of MPP (7.42 cm^3^/g) was similar to that obtained for cladodes powder having 7.50 cm^3^/g (Ayadi et al. 2009), but higher than that recorded for wheat flour accounting for 5.92 cm^3^/g (Adegunwa et al. 2019).

**TABLE 1:** Physico‐chemical properties (A) of *Cucumis melo* L. peel powder and color characteristics (B) of wheat flour and melon peel powder.

(A)	
	Melon peel powder
Moisture (%)	7.76 ± 0.08
Protein (%)	7.63 ± 0.03
Lipid (%)	2.37 ± 0.09
Ash (%)	3.98 ± 0.19
Total carbohydrate (%)	78.26 ± 0.15
Total dietary fiber (%)	40.84 ± 0.07
Bulk density (g/mL)	0.51 ± 0.01
Powder swelling (cm^3^/g)	7.42 ± 0.05
Water solubility index, WSI (%)	32.08 ± 0.11
Water holding capacity (g water/g DM)	5.77 ± 0.12
Oil binding capacity (g fat/g DM)	2.35 ± 0.04

(B)		
	Wheat flour	Melon peel flour
Color		
L*	98.31^a^ ± 0.47	68.63^b^ ± 0.14
a*	0.56^a^ ± 0.03	−2.36^b^ ± 0.07
b*	12.50^a^ ± 0.36	30.19^b^ ± 0.09

*Note:* All the values represent the average ± standard deviation of triplicate experiments. Different lowercase letters within a column indicate significant differences (*p* < 0.05) between the different samples.

Abbreviation: DM, dry matter.

The water solubility index (32.08%) of melon peel powder was higher compared with that of wheat flour (27.49%) as reported by Adegunwa et al. (2019). Water holding capacity is an essential processing parameter mainly in baking applications (Julianti et al. 2017). Melon peel powder showed water holding capacity of 5.77 g water/g; such value was similar to that of pumpkin peel powder accounting for 5.70 g water/g (Nyam et al. 2013) and lower than that recorded for Sharlyn melon peel flour having 7.70 g water/g (Al‐Sayed and Ahmed 2013). The water absorption capacity is principally due to the high number of hydroxyl groups in the fiber structure which leads to water interaction through hydrogen bonding (Ben Jeddou et al. 2017). Melon peel flour exhibited a comparable oil absorption capacity (2.35 g oil/g) with that obtained for Sharlyn melon peel flour (2.24 g oil/g; Al‐Sayed and Ahmed 2013) and watermelon peel flour (2 g oil/g; Romdhane et al. 2024). The oil absorption capacity of the flour depends on several factors related to proteins (amino acid composition, surface polarity, hydrophobicity) (Silva et al. 2024). High oil absorption capacity is often required in baked foods, since oil can act as a flavor retainer and enhance the flavor of the food. In addition, it contributes to extending the shelf life in bakery products where oil holdings are needed (Gigante et al. 2023). Table 1B displays the CieLab coordinates of MPP and wheat flour. It is clear that the wheat flour was lighter (L*) than MPP; while, the green (a*) and yellow (b*) hues of the MPP were more intense than those obtained for wheat flour. The difference in color attributes between both flours is mainly due to the higher pigment content of MPP which comprises several natural pigments. Indeed, melon peel color is based on different combinations of pigments such as chlorophylls, carotenoids and flavonoids depending on their changed ratios during fruit maturation (Tadmor et al. 2010).

### 3.2 Dough Properties

#### 3.2.1 Alveographic Properties of Doughs

The alveographic characteristics of wheat flour doughs (control and MPP‐enriched flours at 10%, 20%, and 30% levels) are shown in Table 2. As seen, the wheat flour substitution with MPP has a significant (*p* < 0.05) effect on tenacity (*P*), extensibility (*L*) and deformation energy (*W*). Indeed, the tenacity (*P*) which illustrates the ability of the dough to retain gas, raised with MPP concentration and the maximal effect being displayed with the addition of 30% MPP in the blend. This could be attributed to the interaction between MPP fibers and wheat flour proteins as reported by Sudha et al. (2007). The dough extensibility (*L*) which is an indication of its handling characteristics, decreased by the addition of MPP. Remarkably, incorporating the MPP at concentrations ranging from 10% to 30% into the wheat flour affects other alveographic parameters of the dough, such as the *P*/*L* ratio and the deformation energy (*W*). In fact, compared to the dough control (*P*/*L* = 0.71 and *W* = 150.35 × 10^−4^ J), the MPP‐enriched doughs exhibited higher values of *P*/*L* and deformation energy (resistance to deformation) especially at the 30% level. Our results are in agreement with those reported by Romdhane et al. (2024) who noticed a decrease in extensibility (*L*) and an increase in resistance to deformation of doughs prepared by incorporating watermelon peels into wheat flour.

**TABLE 2:** Alveograph characteristics (A), textural properties (cohesion, adhesion, stickiness, and hardness) (B) and color characteristics (C) of dough control (100% wheat flour) and doughs prepared with wheat flour fortified with melon peel powder at 10%, 20%, and 30% levels.

(A)				
	*P* (mm H_2_O)	*L* (mm)	*P*/*L*	*W* (10^−4^ J)
Control	67.67^a^ ± 0.07	91.33^a^ ± 0.11	0.71^a^ ± 0.10	150.35^a^ ± 0.25
10%	74^b^ ± 0.04	72^b^ ± 0.07	1.12^b^ ± 0.03	181^b^ ± 0.15
20%	77.33^b,c^ ± 0.05	67^b,c^ ± 0.09	1.16^b,c^ ± 0.08	198.05^c^ ± 0.16
30%	80^c^ ± 0.08	63.67^c^ ± 0.03	1.22^c^ ± 0.04	200.92^c^ ± 0.11

(B)				
	Cohesion (mm)	Adhesion (N)	Stickiness (N)	Hardness (N)
Control	0.23^a^ ± 0.07	0.26^a^ ± 0.11	0.32^a^ ± 0.10	0.94^a^ ± 0.25
10%	0.33^b^ ± 0.04	0.31^b^ ± 0.07	0.50^b^ ± 0.03	0.71^b^ ± 0.15
20%	0.47^c^ ± 0.05	0.39^c^ ± 0.09	0.66^c^ ± 0.08	0.52^c^ ± 0.16
30%	0.53^c^ ± 0.08	0.46^d^ ± 0.03	1.90^d^ ± 0.04	0.48^c^ ± 0.11

(C)				
	Control	10%	20%	30%
Color				
L*	80.55^a^ ± 0.33	68.46^b^ ± 0.10	63.96^c^ ± 0.36	61.54^d^ ± 0.27
a*	0.22^a^ ± 0.03	−1.24^b^ ± 0.01	−1.23^b^ ± 0.00	−1.70^c^ ± 0.00
b*	17.46^a^ ± 0.17	19.62^b^ ± 0.26	23.99^c^ ± 0.10	24.03^c^ ± 0.28

*Note:* Data are means ± standard deviations values of three replicates. *P*, tenacity; *L*, dough extensibility; *P*/*L*, *P*/*L* ratio; *W*, deformation energy. Different lower case letters indicate significant differences (*p* < 0.05) between the different samples.

#### 3.2.2 Textural Characteristics of Doughs

The textural properties of doughs (cohesion, adhesion, stickiness, and hardness), containing 0%, 10%, 20%, and 30% of melon peel flour were analyzed to assess dough quality and the results are presented in Table 2B. The addition of MPP showed a significant effect (*p* < 0.05) on the textural properties of the doughs. Indeed, all doughs containing MPP showed higher cohesion, adhesion and stickiness compared with the control sample. Moreover, these textural parameters increase as the concentration of MPP rises. As reported by Chen et al. (1988), the change in dough rheological and textural features is especially relevant due to the interaction between fibers and gluten, which impacts the dough mixing traits.

Otherwise, the enriched doughs showed a lower hardness as compared to the dough control, and the dough hardness decreased with increased MPP level in the blend. The decrease in hardness of doughs supplemented with MPP indicated softness in the texture which was mainly related to the functional properties of the MPP used in the formulation. Functional properties refer to several attributes of a flour that can affect its behavior during food preparation (Sergiacomo et al. 2025). Indeed, the water holding capacity of MPP influences the hydration and the texture of the dough, while the oil binding capacity affects the retention and distribution of fats, impacting dough softness. The decrease in hardness of enriched doughs could also be ascribed to the reduction of the gluten content as a result of the partial replacement of wheat flour.

#### 3.2.3 Dough Color

The effect of MPP addition on the dough color was investigated by the evaluation of CieLab color parameters and results are demonstrated in Table 2C. As can be seen, the incorporation of MPP, significantly affected (*p* < 0.05) the color of the dough which became darker, particularly at the 30% level. Indeed, the control dough has a higher L* value (80.55) compared to the doughs supplemented with MPP 61.54 (at 30%) and 68.46 (at 10%). Furthermore, all MPP‐enriched doughs exhibited a green hue marked by negative values of the a* color parameter. Moreover, the dough control presented the lowest value of b* when compared to the samples supplemented with melon peel powder. The same trend was found by Ben Jeddou et al. (2017) who reported that the partial substitution of wheat flour by potato peel powder notably affected the dough color and resulted in a decrease of the lightness and an increase of green (a*) and yellow (b*) hues of the enriched doughs.

### 3.3 Baking Test

#### 3.3.1 Physical Characteristics of Cakes

Table 3A illustrated the physical characteristics of cakes prepared with MPP‐enriched wheat flours. The addition of MPP in cake formulation, has no significant effect on the physical parameters (weight, volume, and density) of enriched cakes in comparison with the control cake.

**TABLE 3:** Effect of melon peel flours addition on physical characteristics (A) and textural properties (hardness, cohesion, springiness, adhesion, chewiness, and breaking force) (B) of cakes prepared with wheat flour fortified with melon peel powder at 10%, 20%, and 30% levels.

(A)			
	Weight (g)	Volume (cc)	Density (g/cc)
Control	140.39^a^ ± 0.14	332.16^a^ ± 0.09	0.425^a^ ± 0.01
10%	146.23^a^ ± 0.03	350^a,b^ ± 0.12	0.417^a^ ± 0.07
20%	148.83^a^ ± 0.02	352.50^a,b^ ± 0.21	0.422^a^ ± 0.15
30%	151.66^a^ ± 0.09	358.33^b^ ± 0.04	0.423^a^ ± 0.09

(B)				
	Control	10%	20%	30%
Hardness (N)	1.67^a^ ± 0.15	1.32^b^ ± 0.09	1.07^c^ ± 0.13	1.04^c^ ± 0.01
Cohesion (mm)	0.37^a^ ± 0.07	0.37^a^ ± 0.11	0.33^a^ ± 0.09	0.30^a^ ± 0.14
Springiness (mm)	6.04^a^ ± 0.13	6.10^a^ ± 0.03	5.73^a^ ± 0.03	5.76^a^ ± 0.07
Adhesion (N)	0.83^a^ ± 0.03	0.60^b^ ± 0.04	0.50^c^ ± 0.07	0.21^d^ ± 0.13
Chewiness (Nmm)	3.92^a^ ± 0.06	3.85^a^ ± 0.04	2.40^b^ ± 0.11	1.05^c^ ± 0.20
Breaking force (N)	0.98^a^ ± 0.17	0.77^b^ ± 0.15	0.62^c^ ± 0.07	0.47^d^ ± 0.08

*Note:* All the values represent the average ± standard deviation of triplicate experiments. Different lowercase letters within a column indicate significant differences (*p* < 0.05) between the different fractions.

These findings are in agreement with those reported by Adegunwa et al. (2019) for cake supplemented with watermelon ( *Citrullus lanatus* ) rind flour.

#### 3.3.2 Texture Characterization of Cakes

The cakes enriched with melon peel powder were subjected to a textural analysis. It is noticeable that texture parameters of the cakes changed as a result of the incorporation of MPP in the blend (Table 3B). Indeed, the hardness, adhesion, chewiness and breaking force of the cakes decreased as the level of melon peel flour increased and enriched cakes became less stiff compared with the control cake. The substitution of wheat flour by MPP at 10%, 20%, and 30% decreased the cake hardness by 20.96%, 35.93%, and 37.73%, respectively. Thus, the texture of the cakes was improved with the addition of MPP. This result is in line with the finding of Naknaen et al. (2016) who noticed a decrease in hardness induced by incorporating watermelon peel powder in the cookies, producing a softer texture. Additionally, the increase of the MPP level led to a decrease in the cake adhesion, chewiness and breaking force. Nevertheless, MPP incorporation has no significant effect (*p* > 0.05) on the cohesion and springiness of the cakes. A similar trend was found with the supplementation of lentil flour to cakes (De la Hera et al. 2012). Thus, it is possible to regard that melon peel flour has good qualities to be used as an improver in bakery. In fact, MPP contributes favorably to the structural integrity and mouthfeel of baked products.

#### 3.3.3 Cakes Color

The effect of melon peel incorporation on the cakes' color is an important factor to decide their acceptability by consumers. The crumb and crust color characteristics of the control cake and enriched cakes were illustrated in Table 4. Regarding cake crust color, fortified cakes were different from. Indeed, the latter exhibited higher L* and a* values in comparison with MPP‐enriched cakes. This is mainly due to differences in the initial color of the ingredients due to their original pigments. In fact, the MPP is darker and more greenish (a* value < 0) than wheat flour. This result would suggest that the effect was more pronounced with increasing MPP levels. In fact, cakes formulated with 30% of MPP gave the lowest a* values and the highest b* crust color values. During baking, Maillard and caramelization reactions take place and are responsible for the final crust color (Purlis 2010). Both proteins and MPP fibers containing free sugars might lead to the promotion of the Maillard and caramelization reactions (Naknaen et al. 2016). Furthermore, it is crucial to note that the incorporation of melon peels in cakes can avoid the use of synthetic colorants since the MPP‐enriched cakes have a natural color of pistachio. Similarly, the cake crumb color of the cakes became darker with increasing levels of MPP. Indeed, the highest L* value was recorded for the control cake compared to all samples supplemented with MPP. The same trend was noted by Romdhane et al. (2024) for bread fortified by watermelon peel powder. Moreover, the increase in MPP level in the formulation led to a decrease in crumb redness (a*) and an increase in cake yellowness (b*) in comparison with the control cake.

**TABLE 4:** Color characteristics of crust cakes (A) and crumb cakes (B) prepared with wheat flour fortified with melon peel powder at 10%, 20%, and 30% levels.

	Control	10%	20%	30%
(A) Crust				
L*	33.26^a^ ± 0.52	27.87^b^ ± 0.69	25.03^c^ ± 0.95	23.64^c^ ± 0.24
a*	11.88^a^ ± 0.11	11.18^b^ ± 0.20	10.88^b,c^ ± 0.43	10.42^c^ ± 0.15
b*	22.72^a^ ± 0.24	23.74^a^ ± 0.84	27.08^b^ ± 0.56	33.24^c^ ± 0.47
(B) Crumb				
L*	40.79^a^ ± 0.83	30.15^b^ ± 0.25	28.72^b,c^ ± 0.56	28.20^c^ ± 0.76
a*	6.48^a^ ± 0.04	6.45^a^ ± 0.44	5.71^a^ ± 0.48	2.84^b^ ± 0.29
b*	21.30^a^ ± 0.64	24.86^b^ ± 0.42	26.23^b^ ± 0.73	33.42^c^ ± 0.60

*Note:* Data are means ± standard deviations values of three replicates. Different lowercase letters within a column indicate significant differences (*p* < 0.05) between the different samples.

#### 3.3.4 Sensory Evaluation

The cake quality assessment by sensory evaluation reflects consumer preferences and aversions. The effects of melon peel flour supplementation on the hedonic sensory features of cakes are shown in Table 5. In general, the flour replacer should preserve or improve the color, taste and texture of the cake as compared to the control formula. Overall acceptability of the enriched cakes with 10% of peel flour was not significantly different when compared to the control cake. Thus, substituting wheat flour with melon peel powder up to 10% maintained the sensory attributes of the product. Indeed, the sensory analysis revealed that the panelists preferred the taste, odor and tenderness of enriched cakes containing 10% MPP, which was acceptable to the sensory panel. However, the cake crumb was dark and less acceptable when 30% of melon peel flour was added to the cakes. In general, results revealed that the sensory attributes were satisfactory for MPP‐enriched cakes with a slight preference among tasters for cakes enriched with 10% MPP. Similarly, Ashoka et al. (2021) reported that cakes enriched with watermelon peel powder at a level of 10% were found to be the most acceptable to the sensory panel. Considering the presence of MPP in the enriched cakes, it was assumed that the partial substitution of wheat flour in the cake formulation had a positive effect on the sensory attributes of cakes.

**TABLE 5:** Sensory evaluation of MPP‐enriched cakes and control cake.

	Color		Odor	Taste	Tenderness	Overall acceptability
	Crust	Crumb				
Control	3.90^a^ ± 0.07	3.77^a^ ± 0.11	3.58^a^ ± 1.10	3.60^a^ ± 0.04	4.43^a^ ± 0.19	3.52^a^ ± 0.87
10%	3.83^a,b^ ± 0.04	3.67^a,b^ ± 0.07	3.54^a^ ± 0.73	3.56^a,b^ ± 0.13	4.37^a^ ± 0.16	3.48^a^ ± 0.82
20%	3.80^a,b^ ± 0.05	3.44^b^ ± 0.09	3.47^a^ ± 0.88	3.47^b^ ± 0.12	4.23^a,b^ ± 0.72	3.35^b^ ± 0.68
30%	3.69^b^ ± 0.08	2.37^c^ ± 0.03	3.39^a^ ± 0.54	3.30^c^ ± 0.20	4.00^b^ ± 0.56	3.29^c^ ± 0.66

*Note:* Data are means ± standard deviations values of three replicates. Different lowercase letters within a column indicate significant dfferences (*p* < 0.05) between the different samples.

## 4 Conclusion

Chemical analysis of melon peel powder reveals a significant content of dietary fiber and protein, highlighting their potential for valorization in food applications. Incorporating melon peel flour into food formulations, particularly baked products, not only enhances the nutritional profile, by increasing fiber and protein content, but also modifies the dough's rheological and textural properties. Alveographic and textural analyses demonstrated an increase in dough tenacity and a corresponding decrease in dough extensibility, indicating structural reinforcement likely due to MPP fiber interactions with the gluten network. In baking trials, partial substitution of wheat flour with MPP improved the texture of cakes, as evidenced by a reduction in hardness and chewiness, while cohesion and springiness remained statistically unaffected. This suggests that MPP contributes positively to the mouthfeel and structural integrity of baked products. Additionally, increasing levels of MPP resulted in darker cake crust and crumb, potentially due to Maillard reactions and higher pigment content from the peel, which may appeal to consumers seeking products with a rustic or artisanal appearance. Beyond cake applications, melon peel powder could serve as a functional ingredient in other baked goods, pasta and high‐fiber snack foods. Its use aligns with sustainability goals by promoting food waste valorization. Future research should explore the bioavailability of nutrients in melon peel‐enriched foods, interactions with other food matrix components, and the effect of processing methods on antioxidant activity and shelf life.

## Author Contributions

**Sana Mallek‐Ayadi:** conceptualization (lead), data curation (lead), formal analysis (equal), investigation (equal), methodology (equal), software (lead), visualization (equal), writing – original draft (lead), writing – review and editing (equal). **Neila Bahloul:** investigation (supporting), methodology (supporting), supervision (equal), validation (equal), writing – review and editing (equal).

## Ethics Statement

All procedures performed in the sensory evaluation involving human participants were in accordance with the guidelines of the Regional Committee of the Protection of Persons (CPP SUD), a body within the Faculty of Medicine of Sfax, Tunisia.

## Consent

Informed consent was obtained from each subject prior to their participation in the study.

## Conflicts of Interest

The authors declare no conflicts of interest.

## Data Availability

All relevant data is included within the manuscript. Any inquiries concerning the findings of this study may be directed to the corresponding author upon request.
